# Assessment of Awake and Sleep Bruxism in Fibromyalgia Patients with Temporomandibular Disorders

**DOI:** 10.3390/jcm15020460

**Published:** 2026-01-07

**Authors:** Davide Alessio Fontana, Salvatore Nigliaccio, Francesca Pusateri, Emanuele Di Vita, Pietro Messina, Enzo Cumbo, Antonio Scardina, Elisabetta Raia, Giuseppe Alessandro Scardina

**Affiliations:** Department of Precision Medicine in Medical, Surgical and Critical Care (Me.Pre.C.C.), University of Palermo, 90127 Palermo, Italy; davidealessiofontana@libero.it (D.A.F.); salvo.nigliaccio@gmail.com (S.N.); francescapusateri24@gmail.com (F.P.); emanueledivita95@gmail.com (E.D.V.); pietro.messina01@unipa.it (P.M.); enzo.cumbo@unipa.it (E.C.); antoscardi213@gmail.com (A.S.); elisaraia28@gmail.com (E.R.)

**Keywords:** fibromyalgia, temporomandibular disorders, bruxism, awake bruxism, sleep bruxism, electromyography (EMG)

## Abstract

**Background/Objectives:** Fibromyalgia (FM) is a chronic pain syndrome often associated with musculoskeletal tenderness, fatigue, and sleep disturbances. Temporomandibular disorders (TMDs) and bruxism are frequently observed comorbidities in patients with FM, yet their objective assessment remains limited. This study aimed to evaluate masticatory muscle activity in patients with fibromyalgia and temporomandibular disorders using both static surface electromyography (sEMG) and a 24 h portable EMG device (Dia-BRUXO^®^). **Methods:** Thirty female patients (mean age 53.6 ± 10.5 years) underwent comprehensive clinical and gnathological evaluations, followed by static EMG recordings of the masseter and temporalis muscles and continuous monitoring of the left masseter over a 24 h period. **Results:** Results revealed a significantly higher number of bruxism episodes during wakefulness (80.9 ± 130.8) compared to sleep (24.0 ± 26.8; *p* < 0.0001). The Masseter Time Index (MTI) and Masseter Work Index (MWI) were also significantly higher during wakefulness (*p* < 0.001), indicating a predominance of daytime masticatory muscle activity. Static sEMG analysis showed generally preserved bilateral muscle symmetry, accompanied by mild imbalances in occlusal load distribution and increased global muscle activation. **Conclusions:** These findings suggest that patients with fibromyalgia and temporomandibular disorders exhibit increased baseline masticatory muscle activity, particularly during wakefulness, possibly reflecting sustained neuromuscular tension. Continuous EMG monitoring appears to provide an objective tool for characterizing bruxism patterns and complements clinical assessment and self-reported data. However, the absence of a control group and the exclusive inclusion of female patients limit the generalizability of the results. Further studies including appropriate comparison groups are needed to clarify the specificity and clinical implications of these findings.

## 1. Introduction

Fibromyalgia (FM) is a chronic pain syndrome characterized by widespread musculoskeletal pain, fatigue, sleep disturbances, and heightened pain sensitivity. In addition to diffuse pain, patients frequently report symptoms such as morning stiffness, headache, cognitive disturbances, and mood alterations, all of which contribute to a substantial reduction in quality of life. The diagnosis is currently based on clinical criteria defined by the American College of Rheumatology, reflecting the absence of specific biomarkers and the still incompletely understood pathophysiology of the condition. Although multiple hypotheses have been proposed, including central sensitization, altered pain modulation, and autonomic dysregulation, FM remains a complex disorder with predominantly symptomatic management strategies [1,2,3].

Beyond generalized musculoskeletal involvement, fibromyalgia is often associated with regional pain syndromes, particularly affecting the craniofacial and cervical districts. Temporomandibular disorders (TMDs) represent one of the most frequently reported comorbid conditions in FM patients. TMDs comprise a heterogeneous group of disorders involving the temporomandibular joint, masticatory muscles, and associated structures, commonly presenting with orofacial pain, functional limitation, joint noises, and headaches.

Epidemiological studies consistently report a significantly higher prevalence of TMDs in individuals with FM compared to the general population, suggesting shared pathophysiological mechanisms rather than a coincidental overlap [4,5,6,7,8,9,10,11,12,13].

A growing body of evidence supports the hypothesis that fibromyalgia-related neuromuscular hyperexcitability and altered pain processing may predispose patients to dysfunctions of the stomatognathic system. Increased muscle tone, reduced pain thresholds, and impaired motor control, which are well-documented features of FM, may contribute to sustained masticatory muscle activity and to the development or exacerbation of myogenous TMDs. Within this framework, bruxism has attracted increasing attention as a potential behavioral and neuromuscular manifestation linking FM and TMD.

Bruxism is currently defined as a repetitive masticatory muscle activity characterized by clenching or grinding of the teeth and/or by bracing or thrusting of the mandible, and is classified according to its circadian phenotype into sleep bruxism and awake bruxism.

While sleep bruxism is considered a sleep-related movement disorder with complex neurophysiological regulation, awake bruxism is more closely associated with psychosocial factors such as stress, anxiety, and sustained attention, often occurring without conscious awareness. Despite its high estimated prevalence in adult populations, the true burden of bruxism remains difficult to quantify due to the limitations of self-reported assessments and the lack of standardized objective diagnostic criteria [14,15,16,17].

The relationship between bruxism and TMD has been widely investigated, with several systematic reviews suggesting a positive association, albeit with heterogeneous methodological quality and diagnostic definitions. Importantly, both conditions appear to share common biological, psychological, and environmental risk factors, including stress-related muscle hyperactivity and altered motor control.

In patients with fibromyalgia, these mechanisms may be amplified by central sensitization and by a generalized increase in baseline muscle activity, potentially favoring the persistence of parafunctional behaviors [18,19,20,21].

Current diagnostic frameworks emphasize the need for a multimodal approach to bruxism assessment, combining clinical examination, self-reported data, and instrumental evaluation. Among available objective tools, electromyography (EMG) represents one of the most sensitive methods for detecting masticatory muscle activity. Traditional surface EMG recordings performed under standardized conditions allow the assessment of muscle symmetry, coordination, and occlusal balance, but provide only a snapshot of neuromuscular behavior.

In contrast, recent advances in portable EMG technology have enabled continuous 24 h monitoring of masticatory muscle activity under real-life conditions, offering valuable insight into the temporal distribution and intensity of both awake and sleep bruxism episodes [22,23,24,25].

In patients with fibromyalgia and TMD, the combined use of static surface EMG and ambulatory EMG monitoring may be particularly informative. Static recordings can characterize baseline neuromuscular balance and occlusal function, while prolonged monitoring captures sustained or repetitive muscle activity that may not be perceived by patients and may differ substantially between wakefulness and sleep. This integrative approach addresses the limitations of subjective reporting and may help clarify whether masticatory muscle hyperactivity in FM reflects predominantly nocturnal phenomena, daytime behavioral patterns, or a continuous state of increased neuromuscular tone.

Therefore, the aim of the present study was to evaluate masticatory muscle activity in patients with fibromyalgia and temporomandibular disorders using both static surface electromyography and a 24 h portable EMG system.

By comparing awake and sleep bruxism patterns, this study seeks to contribute to a more precise characterization of parafunctional activity in this complex clinical population and to support a more comprehensive, instrument-based diagnostic approach.

## 2. Materials and Methods

### 2.1. Participants

The study involved 30 female patients consecutively recruited at the Department of Precision Medicine in Medical, Surgical and Critical Care (Me.Pre.C.C.), University of Palermo. All participants had a confirmed diagnosis of fibromyalgia according to the American College of Rheumatology (ACR) criteria and were referred for clinical evaluation of temporomandibular disorders.

Only female patients were included in the present sample. This choice reflects the well-documented higher prevalence of fibromyalgia in women and the demographic characteristics of patients referred to the study center during the recruitment period.

However, the exclusive inclusion of female participants should be acknowledged as a limitation, as it restricts the generalizability of the findings to male patients, in whom pain perception, muscle activity, and parafunctional behaviors may differ.

The mean age of the sample was 53.6 ± 10.5 years. Rheumatologic assessment revealed a high severity profile of fibromyalgia symptoms within the studied population: approximately 27% of patients (8/30) were classified in the “severe” category, while the remaining 73% (22/30) fell into the “very severe” category. No participants were classified in lower severity ranges. This distribution likely reflects a referral bias toward patients with more advanced or clinically complex fibromyalgia, as commonly observed in tertiary care settings. Consequently, the results should be interpreted with caution when extrapolating to patients with milder forms of the disease.

Regarding pharmacological management, 22 out of 30 patients (approximately 73%) reported the use of anti-inflammatory drugs or corticosteroids, 11 patients (approximately 37%) were taking muscle relaxants, and 10 patients (approximately 33%) reported the use of non-smoked cannabis. Medication use was recorded during anamnesis but was not controlled or stratified in the analysis, representing a potential confounding factor. The influence of pharmacological treatment on masticatory muscle activity could not be specifically assessed and should therefore be considered a methodological limitation of the present study.

All participants provided written informed consent and were informed of their right to withdraw from the study at any time without consequences.

### 2.2. Materials

#### 2.2.1. Static Surface Electromyography [25]

Static electromyographic recordings were performed using a surface EMG system designed for the assessment of masticatory muscle activity and occlusal balance (Kinelock^®^, 4T–QuattroTi, Cisalgo, Italy). The device allows bilateral recording of the masseter and anterior temporalis muscles and provides quantitative indices describing muscle symmetry, coordination, and global activation during standardized clenching tasks.

Surface electrodes were positioned according to the manufacturer’s guidelines after careful skin preparation to minimize impedance. Real-time signal visualization was used to verify electrode placement and signal quality prior to data acquisition. The EMG system automatically computes validated indices commonly used in gnathological assessment, including symmetry coefficients, occlusal load distribution, and global muscle activation parameters, which were subsequently analyzed in the present study.

Detailed technical specifications of the device, including electrode characteristics, sampling frequency, and signal processing parameters, are provided in the article.

#### 2.2.2. Portable 24-Hour Electromyographic Monitoring [26]

Continuous ambulatory monitoring of masticatory muscle activity was performed using a portable EMG-Holter device (Dia-BRUXO^®^, Biotech-Novations, Sanremo, Italy). The system enables prolonged recording of electromyographic activity under real-life conditions and provides an objective assessment of both awake and sleep bruxism.

For each participant, the device recorded the activity of the left masseter muscle using a disposable bipolar surface electrode. The choice of a single muscle was based on the device design and on previous validation studies demonstrating the suitability of masseter activity as a proxy for overall bruxism-related muscle activation. The left side was selected to standardize recordings across participants and reduce inter-subject variability related to electrode placement.

Before the start of monitoring, participant-specific information was entered into the dedicated software, and signal quality was checked. The device continuously recorded muscle activity for 24 h, after which the data were downloaded and analyzed. The software automatically identified bursts of muscle activity and classified them as awake or sleep bruxism episodes based on the participant’s circadian rhythm, generating quantitative indices related to duration, workload, and overall bruxism severity.

Technical details regarding signal amplification, filtering, and processing algorithms are reported in the article.

#### 2.2.3. Methodological Considerations and Device Limitations

Some methodological limitations related to the electromyographic devices should be acknowledged. The portable EMG system records activity from a single masseter muscle and therefore does not capture bilateral or temporalis muscle involvement, which may lead to an underestimation of total masticatory muscle activity, particularly in subjects with asymmetric parafunctional patterns. However, the use of a standardized recording site allowed consistent intra-sample comparisons between awake and sleep conditions.

Moreover, surface EMG recordings are inherently sensitive to factors such as skin impedance, subcutaneous tissue thickness, electrode positioning, and movement artifacts. To mitigate these effects, standardized skin preparation, careful electrode placement, and real-time signal verification were adopted. Nonetheless, residual variability cannot be completely excluded and represents an intrinsic limitation of non-invasive EMG techniques.

### 2.3. Study Protocol

Each participant underwent a standardized evaluation protocol conducted by the same clinical team to minimize inter-operator variability. The protocol consisted of the following sequential steps.

First, a comprehensive medical and dental history was collected, with particular attention to fibromyalgia management, temporomandibular disorder-related symptoms, and self-reported parafunctional habits. This was followed by a full gnathological examination, including functional assessment of mandibular movements and palpation of the masticatory and cervical muscles to evaluate pain and tenderness.

Static surface electromyographic recordings were then performed using the Kinelock^®^ system. Recordings were acquired under three conditions: one trial with cotton rolls positioned between the dental arches and two consecutive trials without cotton rolls. The recording with cotton rolls was used as a reference condition to minimize occlusal influences and to assess baseline neuromuscular symmetry. The two trials without cotton rolls were performed to improve the stability of the measurements and to reduce the potential impact of transient variability related to patient adaptation or initial unfamiliarity with the task. Although formal intra-session reliability analysis was not conducted, repeated recordings were used to enhance the robustness of the collected data.

During static EMG acquisition, participants were instructed to perform a maximum voluntary clench in a seated position, maintaining a natural head posture. No external force feedback system was used to standardize clenching intensity; therefore, maximum voluntary contraction was guided by verbal instructions and real-time signal monitoring.

This approach reflects common clinical practice but should be considered a methodological limitation, as inter-individual differences in clenching effort may have influenced absolute EMG amplitudes.

After completion of the static EMG recordings, the Dia-BRUXO^®^ portable electromyographic device was applied. Participants were instructed to wear the device continuously for 24 h during their usual daily activities and sleep, avoiding removal except when strictly necessary. On the following day, the device was returned for data extraction and analysis using the manufacturer’s dedicated software.

### 2.4. Statistical Analysis

All data collected from the static and ambulatory electromyographic recordings were entered into a dedicated spreadsheet (Microsoft Excel 2021, Los Angeles, CA, USA) and subsequently analyzed using XLSTAT software (version 2025.1, Addinsoft, New York, NY, USA).

Descriptive statistics were calculated for all variables, including means, standard deviations, medians, and ranges, as appropriate. The number of bruxism episodes recorded over 24 h was analyzed separately for wakefulness and sleep periods.

Prior to inferential analysis, data distribution was explored using graphical inspection and tests for normality (Shapiro–Wilk test). Given the marked inter-individual variability observed in several parameters, with wide ranges and skewed distributions suggested by differences between mean and median values, both parametric and non-parametric approaches were considered.

The comparison between the number of awake and sleep bruxism episodes was performed using a paired *t*-test. This choice was based on the within-subject design of the comparison and on the robustness of the *t*-test to moderate deviations from normality in paired data. Nevertheless, the results should be interpreted with caution considering the observed variability.

For the analysis of Dia-BRUXO^®^ indices (Masseter Time Index, Masseter Work Index, Bruxism Time Index, Bruxism Work Index, and Bruxism Personal Index), comparisons between awake and sleep conditions were performed using the Mann–Whitney U test, as these indices did not meet normality assumptions (Table 1).

Static EMG parameters obtained from the Kinelock^®^ system were analyzed descriptively and compared with reference values provided by the manufacturer and validated in the literature, without inferential comparison to external control groups (Table 2).

No formal correction for multiple comparisons was applied, given the exploratory nature of the study and the limited sample size. Accordingly, the statistical findings should be interpreted as hypothesis-generating rather than confirmatory.

The level of statistical significance was set at *p* < 0.05 for all analyses.

## 3. Results

### 3.1. Intraoral and Extraoral Clinical Examination

#### 3.1.1. Intraoral Clinical Examination

The intraoral examination revealed a high prevalence of posterior dental alterations. Posterior edentulism was observed in 70% of patients (21/30), while posterior prosthetic rehabilitations were present in approximately 47% of cases (14/30). These findings suggest a frequent alteration of posterior occlusal support within the study population, which may contribute to functional imbalance of the stomatognathic system.

Dental attrition compatible with parafunctional activity was detected in 57% of patients. Furthermore, 67% of participants (20/30) self-reported bruxism during anamnesis, with 43% (13/30) indicating the presence of both awake and sleep bruxism. These data confirm a high prevalence of occlusal parafunctions in this cohort.

#### 3.1.2. Muscle Examination

Clinical palpation revealed widespread muscle tenderness involving both masticatory and cervical muscle groups. The lateral pterygoid muscle showed the highest prevalence of pain, affecting 97% of patients (29/30). Masseter muscle tenderness was present in 93% of cases (28/30), followed by the temporalis muscle in 70% (21/30).

Cervical muscle involvement was also frequent, with tenderness of the sternocleidomastoid and trapezius muscles reported in 83% of patients (25/30 for each muscle). Although unilateral pain was observed in a subset of patients across different muscle groups, the overall pattern was characterized by diffuse bilateral involvement, consistent with the generalized pain sensitivity typical of fibromyalgia.

#### 3.1.3. Mandibular Kinematics and Joint Sounds

Alterations in mandibular movement were observed in 87% of patients (26/30). Deviations during mouth opening were the most common finding, frequently exhibiting an S-shaped pattern, in some cases associated with posterior crossbite. Additional deviations were observed during closing movements or during both opening and closing phases.

Joint sounds during mandibular movement were reported by 80% of patients (24/30), predominantly in the form of clicking, suggesting the presence of mechanical components of temporomandibular disorders within the studied population.

#### 3.1.4. Differential Clinical Tests

Differential diagnostic tests were performed to distinguish between muscular and articular components of temporomandibular pain. The End Feel test suggested a predominantly muscular origin in 33% of patients (10/30) and an articular origin in 13% (4/30).

Similarly, the Bite test indicated a muscular response in 60% of cases (18/30), whereas an articular response was observed in 6% (2/30). Overall, these findings support a prevailing myogenous component of TMD-related pain in this cohort (Table 3 and Table 4).

The mean number of bruxism episodes recorded over the 24 h monitoring period differed markedly between wakefulness and sleep (Table 3). During wakefulness, participants exhibited a mean of 80.9 ± 130.8 bruxism episodes (median = 39; range: 3–668), whereas during sleep the mean number of episodes was 24.0 ± 26.8 (median = 18.5; range: 0–123).

The comparison between awake and sleep conditions revealed a statistically significant difference, with a substantially higher frequency of bruxism episodes occurring during wakefulness (paired *t*-test, *p* < 0.0001).

These findings indicate a clear predominance of daytime masticatory muscle activity in the studied population, suggesting that parafunctional behaviors in patients with fibromyalgia and temporomandibular disorders may be more strongly expressed during wakefulness than during sleep.

Quantitative indices derived from the 24 h portable electromyographic monitoring are reported in Table 3 and Table 4. Comparisons between awake and sleep conditions were performed for all Dia-BRUXO^®^ parameters.

The Masseter Time Index (MTI) and Masseter Work Index (MWI) were significantly higher during wakefulness compared with sleep (*p* < 0.001 for both). These results indicate that, in the studied population, masseter muscle activity during wakefulness occupied a greater proportion of the total recording time and was associated with a higher cumulative muscular workload.

**Figure 1:**
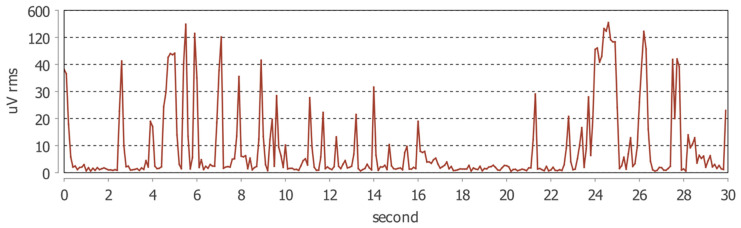
Example of a 30 s electromyographic recording from the Dia-Bruxo^®^ device showing RMS amplitude (µV) of the left masseter muscle. The peaks correspond to bursts of masticatory muscle activity detected during an awake bruxism episode.

**Figure 2:**
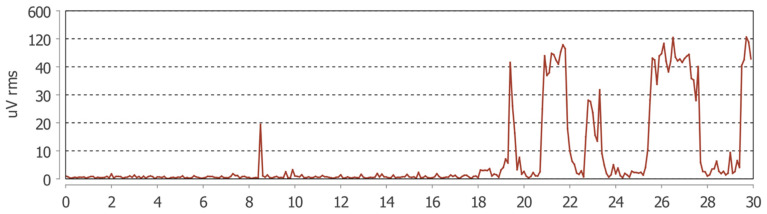
Example of a 30 s electromyographic recording from the Dia-Bruxo^®^ device showing RMS amplitude (µV) of the left masseter muscle. The peaks correspond to bursts of masticatory muscle activity detected during a sleep bruxism episode.

In contrast, no statistically significant differences were observed between awake and sleep conditions for the Bruxism Time Index (BTI), Bruxism Work Index (BWI), or Bruxism Personal Index (BPI), although all three parameters consistently showed higher mean values during wakefulness. This pattern suggests that the primary circadian difference lies in the temporal extent and overall workload of masticatory muscle activity rather than in the intensity of individual bruxism episodes (Figure 1 and Figure 2).

### 3.2. Kinelock Indices

Static surface electromyographic parameters obtained with the Kinelock^®^ system are summarized in Table 5 and were interpreted in relation to reference values reported in the literature.

The Percentage Overlapping Coefficient for the anterior temporalis muscles (POC TA) and for the masseter muscles (POC MM) showed mean values of 83.44 ± 12.17% and 82.54 ± 13.19%, respectively, indicating an overall good level of bilateral neuromuscular symmetry during clenching. Although individual variability was present, most values were close to or within the physiological reference range.

The Barycenter of Force (BAR) exhibited a mean value of 84.99 ± 12.03%, slightly below the optimal reference range (90–100%), suggesting a mild imbalance in occlusal load distribution at the group level. In contrast, the Torsional Index (TORS) demonstrated a mean value of 90.2 ± 7.31%, consistent with a generally balanced torsional pattern between contralateral muscle groups.

The Impact Index (IMPACT), reflecting the overall magnitude of muscular activation during clenching, showed a mean value of 126.73 ± 63.72%, exceeding the physiological reference interval (85–115%). Notably, this parameter displayed a wide standard deviation, indicating substantial inter-individual variability in global muscle activation within the sample.

**Figure 3:**
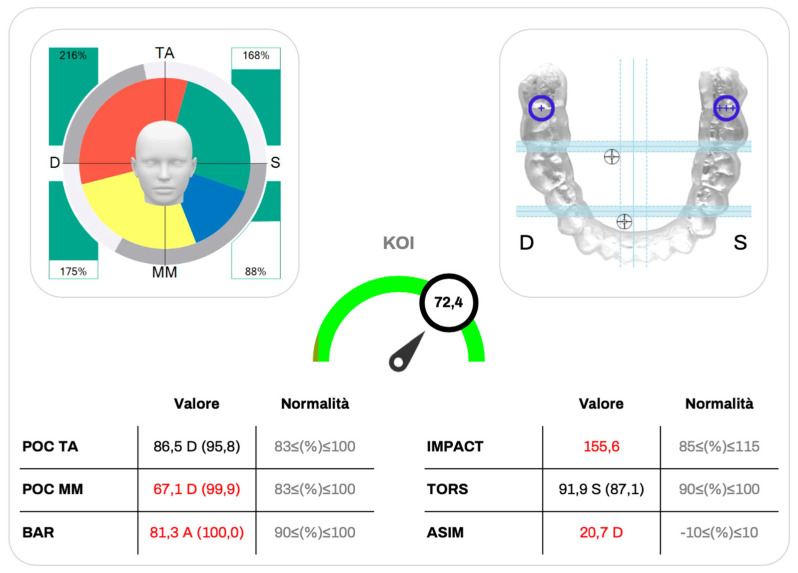
Example of Kinelock^®^ sEMG report showing masticatory muscle activity and occlusal balance. The circular diagram illustrates temporalis and masseter activation symmetry, while the occlusal map displays the barycenter of force. Quantitative indices (POC, BAR, IMPACT, TORS, ASIM, KOI) summarize neuromuscular coordination and occlusal stability.

**Figure 4:**
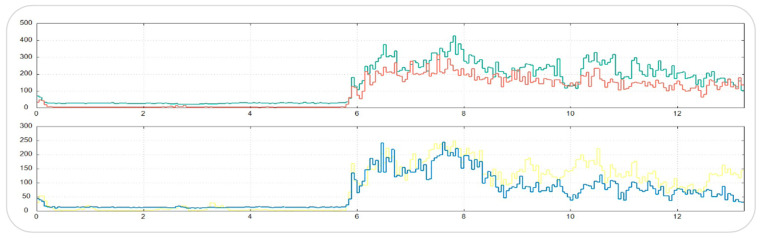
Raw sEMG traces from the Kinelock^®^ system showing real-time electrical activity of the right and left temporalis and masseter muscles during clenching. The graphs illustrate bilateral muscle activation patterns used for subsequent computation of symmetry and occlusal indices. The two different colors in the graph represent simultaneously recorded sEMG signals from different sides of the same muscle group. The red trace indicates the right anterior temporalis muscle, while the green trace represents the left anterior temporalis muscle. The yellow trace indicates the right masseter muscle, and the blue trace represents the left masseter muscle.

The Asymmetry Index (ASIM) presented a mean value close to zero (0.35 ± 19.57%), although with a broad dispersion of values, reflecting heterogeneous lateral activation patterns across participants without a consistent directional bias. Finally, the Kinematic Occlusal Index (KOI) showed a mean value of 75.81 ± 17.6%, positioned near the upper limit of the optimal reference range (70–80%), suggesting an overall satisfactory level of occlusal stability and neuromuscular efficiency despite individual variability (Figure 3 and Figure 4).

## 4. Discussion

The present study investigated masticatory muscle activity in a cohort of fibromyalgia patients with temporomandibular disorders using both static surface electromyography and continuous 24 h EMG monitoring. This combined approach allowed the evaluation of masticatory muscle behavior under standardized conditions as well as during daily activities and sleep, providing objective information on both awake and sleep bruxism patterns.

The main finding of this study is the significantly higher number of bruxism episodes observed during wakefulness compared with sleep. This result is supported by the Dia-BRUXO^®^ indices, which showed higher Masseter Time Index (MTI) and Masseter Work Index (MWI) values during the daytime. These data indicate that, in the present sample, masticatory muscle activity during wakefulness occupies a larger proportion of time and is associated with a greater cumulative muscular workload. Similar observations have been reported in previous studies suggesting that awake bruxism may represent a relevant source of prolonged muscle activation, particularly in patients exposed to chronic stress or pain conditions.

Although fibromyalgia is characterized by widespread pain and altered pain processing, the predominance of awake bruxism observed in this study should not be interpreted as a feature specific to fibromyalgia. Rather, it may reflect a pattern of sustained neuromuscular activation that becomes more evident in this population due to reduced pain thresholds and limited adaptive capacity.

For this reason, the hypothesis that awake bruxism represents a widespread behavioral phenotype should be considered with caution and regarded as a possible direction for future research rather than a definitive conclusion.

The static EMG findings obtained with the Kinelock^®^ system showed an overall good bilateral symmetry of the masseter and temporalis muscles, as indicated by the Percentage Overlapping Coefficients. However, some indices suggested subtle functional alterations. In particular, the Impact Index exceeded the physiological reference range and showed wide inter-individual variability. This finding suggests that, despite preserved symmetry, fibromyalgia patients with TMD may present an increased level of global muscle activation during clenching, which could contribute to muscle fatigue and pain persistence.

An important aspect emerging from this study is the discrepancy between self-reported bruxism and objectively recorded muscle activity. Several patients who showed clear EMG evidence of awake or sleep bruxism were unaware of any parafunctional behavior, while others reported bruxism that was not confirmed instrumentally.

These inconsistencies highlight the limited reliability of self-reported bruxism, particularly in patients with chronic pain syndromes, and support the use of objective tools for assessment. In fibromyalgia patients, factors such as stress, pain catastrophization, and somatosensory amplification may further reduce the accuracy of subjective reporting.

Recent evidence supports the close relationship between fibromyalgia severity and orofacial involvement. In this regard, Puleio et al. [27] reported that higher Fibromyalgia Assessment Status scores are associated with an increased probability of developing orofacial disorders. This observation is consistent with the present findings and supports the hypothesis that masticatory muscle hyperactivity and TMD symptoms may represent part of a broader clinical spectrum in fibromyalgia, rather than isolated comorbid conditions.

The present study has several limitations. The exclusive inclusion of female patients limits the generalizability of the results, and the absence of a control group prevents direct comparison with healthy subjects or fibromyalgia patients without TMD.

In addition, pharmacological treatments were recorded but not controlled for, and the portable EMG device recorded activity from a single masseter muscle, potentially underestimating bilateral or asymmetric activity. These factors should be considered when interpreting the results.

Despite these limitations, the use of continuous 24 h electromyographic monitoring represents a strength of the study. By providing objective information on the temporal distribution and workload of masticatory muscle activity, EMG monitoring may help improve the clinical assessment of bruxism and TMD in fibromyalgia patients and support a more integrated, multidisciplinary approach to management.

## 5. Conclusions

The present study provides objective evidence of increased masticatory muscle activity in patients with fibromyalgia and temporomandibular disorders, with a clear predominance during wakefulness.

Continuous electromyographic monitoring revealed a higher number of daytime bruxism episodes as well as increased Masseter Time Index and Masseter Work Index values during wakefulness, suggesting sustained diurnal muscle activation rather than a purely sleep-related phenomenon.

Static surface electromyography further indicated the presence of altered neuromuscular function, characterized by elevated global muscle activation and mild imbalances in occlusal load distribution, despite generally preserved bilateral symmetry.

These findings support the hypothesis that fibromyalgia is associated with a state of increased baseline muscle activity, which may become clinically evident in the stomatognathic system due to its high functional demands.

However, the absence of a control group and the exclusive inclusion of female patients limit the generalizability of the results. In addition, pharmacological treatments and methodological constraints related to electromyographic assessment should be considered when interpreting the data.

Therefore, the observed predominance of awake bruxism cannot be considered specific to fibromyalgia but may reflect a broader pattern of stress-related or pain-associated neuromuscular behavior.

Despite these limitations, the combined use of static and dynamic electromyography represents a valuable approach for the objective assessment of masticatory muscle activity. In clinical practice, prolonged EMG monitoring may help identify underrecognized parafunctional behaviors and support a more comprehensive, multidisciplinary management of patients with fibromyalgia and temporomandibular disorders. Further studies, including appropriate control groups and less severe fibromyalgia phenotypes, are needed to clarify the specificity and clinical implications of these findings.

## Figures and Tables

**Table 1:** Dia-BRUXO Parameters.

Index	Full Name	Definition	Unit	Represents
**MTI**	Masseter Time Index	Percentage of time showing masseter muscle activity with respect to the total EMG-Holter recording duration.	%	Temporal extent of overall masseter activity.
**MWI**	Masseter Work Index	Percentage of muscular work generated by the masseter muscle relative to the potential work that would have been produced if the maximum recorded power peak had been continuously maintained for the entire EMG-Holter monitoring period.	%	Overall muscular workload or functional performance of the masseter.
**BTI**	Bruxism Time Index	Percentage of time characterized by bruxism episodes during the entire EMG-Holter monitoring period.	%	Temporal extent of bruxism activity.
**BWI**	Bruxism Work Index	Percentage of muscular work performed during bruxism episodes relative to the potential work that would have been produced if the highest recorded power peak had been continuously maintained throughout all bruxism episodes.	%	Muscular effort or intensity of bruxism.
**BPI**	Bruxism Personal Index	Composite indicator derived from BTI and BWI, representing the individual’s overall level of bruxism, integrating both time and intensity parameters.	Dimensionless	Global bruxism severity

**Table 2:** Kinelock Parameters.

Index	Full Name	Definition	Unit	Represents
**POC TA**	Percentage Overlapping Coefficient—Temporalis Anterior	Quantifies the bilateral symmetry of the anterior temporalis muscles during clenching, expressed as a percentage of overlap between left and right EMG activity.	%	Neuromuscular coordination and functional symmetry of the temporalis muscles.
**POC MM**	Percentage Overlapping Coefficient—Masseter Muscles	Quantifies the bilateral symmetry of the masseter muscles during clenching, expressed as a percentage of overlap between left and right EMG activity.	%	Neuromuscular coordination and balance of the masseter muscles.
**BAR**	Barycenter of Force	Indicates the occlusal force center of gravity, calculated from the relative activation of right and left masseter and temporalis muscles.	%	Distribution of occlusal load between dental arches.
**IMPACT**	Impact Index	Represents the overall magnitude of muscular activity during clenching, derived from the sum of EMG amplitudes relative to a normative reference.	%	Global muscle activation level/functional tone.
**TORS**	Torsional Index	Expresses the degree of torsional balance between contralateral pairs of muscles (right temporalis + left masseter vs. left temporalis + right masseter).	%	Bilateral torsional control and occlusal coordination.
**ASIM**	Asymmetry Index	Measures the lateral deviation of total EMG activity between right and left muscle groups.	%	Direction and magnitude of functional asymmetry.
**KOI**	Kinematic Occlusal Index	Composite index derived from BAR, POC, and TORS values, expressing the overall occlusal stability and neuromuscular equilibrium.	%	Global occlusal stability and neuromuscular efficiency.

**Table 3:** Number of Bruxism Episodes per 24 h.

Mean	SD	Median	Range Min	Range Max	*t*-Test	*p*-Value
**Sleep**	23.96667	26.76009	18.5	0	123	**<0.0001**
**Awake**	80.93333	130.8484	39	3	668	

**Table 4:** Dia-BRUXO Indices.

	Awake					Sleep						
	Min	Max	Mean	SD	Median	Min	Max	Mean	SD	Median	Trend	*p*-Value
**MTI**	6.90	96.99	32.09	26.71	21.87	0.37	99.19	14.83	25.04	4.45	↓	**<0.0001**
**MWI**	1.22	17.13	5.52	4.36	3.63	0.09	12.58	1.98	2.95	0.88	↓	**<0.0001**
**BTI**	0.03	5.12	0.89	1.19	0.44	0.00	1.10	0.35	0.29	0.30	↓	<0.082
**BWI**	0.04	3.41	0.65	0.84	0.35	0.00	0.77	0.29	0.25	0.25	↓	<0.12
**BPI**	0.03	4.55	0.81	1.06	0.42	0.00	0.88	0.33	0.26	0.30	↓	<0.082

**Table 5:** Static surface electromyographic parameters obtained with the Kinelock® system.

	Min	Max	Mean	SD	Reference Values
**POC TA**	47.1	97.2	83.44	12.17	83 ≤ (%) ≤ 100
**POC MM**	35.25	93.6	**82.54**	13.19	83 ≤ (%) ≤ 100
**BAR**	40.2	94.8	**84.99**	12.03	90 ≤ (%) ≤ 100
**IMPACT**	43.4	296.9	**126.73**	63.72	85 ≤ (%) ≤ 115
**TORS**	56.9	95.0	90.2	7.31	90 ≤ (%) ≤ 100
**ASIM**	−41.4	53.15	0.35	19.57	(−10) ≤ (%) ≤ 10
**KOI**	17.4	92.8	75.81	17.6	70 ≤ (%) ≤ 80

## Data Availability

The raw data supporting the conclusions of this article will be made available by the authors on request.

## Abbreviations

The following abbreviations are used in this manuscript:

FM	Fibromyalgia
TMD/TMDs	Temporomandibular Disorder(s)
TMJ	Temporomandibular Joint
sEMG	Surface Electromyography
EMG	Electromyography
ACR	American College of Rheumatology
RMS	Root Mean Square
CODE	Concentric Electrode (proprietary system)
POC	Percent of Overlapping Coefficient
POC TA	Percent Overlapping Coefficient—Temporalis Anterior
POC MM	Percent Overlapping Coefficient—Masseter Muscles
BAR	Barycenter of Force
IMPACT	Impact Index
TORS	Torsional Index
ASIM	Asymmetry Index
KOI	Kinematic Occlusal Index
MTI	Masseter Time Index
MWI	Masseter Work Index
BTI	Bruxism Time Index
BWI	Bruxism Work Index
BPI	Bruxism Personal Index
SCM	Sternocleidomastoid Muscle
